# Oriented Three-Dimensional Skeletons Assembled by Si_3_N_4_ Nanowires/AlN Particles as Fillers for Improving Thermal Conductivity of Epoxy Composites

**DOI:** 10.3390/polym15224429

**Published:** 2023-11-16

**Authors:** Baokai Wang, Shiqin Wan, Mengyang Niu, Mengyi Li, Chang Yu, Zheng Zhao, Weiwei Xuan, Ming Yue, Wenbin Cao, Qi Wang

**Affiliations:** 1 School of Materials Science and Engineering, University of Science and Technology Beijing, Beijing 100083, China; 2 School of Energy and Environmental Engineering, University of Science and Technology Beijing, Beijing 100083, China; 3 School of Civil and Resource Engineering, University of Science and Technology Beijing, Beijing 100083, China; yueming0101@163.com

**Keywords:** silicon nitride nanowires, thermal interface materials, ice template method, thermal conductivity

## Abstract

With the miniaturization of current electronic products, ceramic/polymer composites with excellent thermal conductivity have become of increasing interest. Traditionally, higher filler fractions are required to obtain a high thermal conductivity, but this leads to a decrease in the mechanical properties of the composites and increases the cost. In this study, silicon nitride nanowires (Si_3_N_4_NWs) with high aspect ratios were successfully prepared by a modified carbothermal reduction method, which was further combined with AlN particles to prepare the epoxy-based composites. The results showed that the Si_3_N_4_NWs were beneficial for constructing a continuous thermal conductive pathway as a connecting bridge. On this basis, an aligned three-dimensional skeleton was constructed by the ice template method, which further favored improving the thermal conductivity of the composites. When the mass fraction of Si_3_N_4_NWs added was 1.5 wt% and the mass fraction of AlN was 65 wt%, the composites prepared by ice templates reached a thermal conductivity of 1.64 W·m^−1^·K^−1^, which was ~ 720% of the thermal conductivity of the pure EP (0.2 W·m^−1^·K^−1^). The enhancement effect of Si_3_N_4_NWs and directional filler skeletons on the composite thermal conductivity were further demonstrated through the actual heat transfer process and finite element simulations. Furthermore, the thermal stability and mechanical properties of the composites were also improved by the introduction of Si_3_N_4_NWs, suggesting that prepared composites exhibit broad prospects in the field of thermal management.

## 1. Introduction

The miniaturization and high degree of integration of electronic devices leads to a sharp increase in heat generation during operation. If timely and effective heat dissipation is not achieved, the operational efficiency and service life of the equipment are seriously affected [1,2,3]. The high-performance thermal interface materials (TIMs) that are used between a heat source and a heat sink have received widespread attention due to their importance in improving the thermal efficiency of electronic equipment. Traditional polymers have the advantage of being light weight, involving easy processing, and having superior electrical insulation; yet, the low intrinsic thermal conductivity of polymers strongly hinders their actual applications [4]. In order to solve this problem, the most commonly used method is to take the polymer as the matrix and then add fillers with high thermal conductivity to prepare composite materials [5,6]. Up to now, numerous filler types have been reported, including metal (e.g., silver, copper), carbon materials (e.g., carbon nanotubes, carbon fibers) and ceramics (e.g., nitrides, oxides) [7,8,9,10]. Although both metal and carbon materials have high thermal conductivity, the excellent electrical conductivity severely limits their use in applications requiring high insulation. In contrast, ceramic materials have high thermal conductivity and low electrical conductivity, thus attracting more attention and research interest.

Among ceramics materials, aluminum nitride (AlN) is one of the ideal fillers for the preparation of TIMs due to its high theoretical thermal conductivity of 320 W∙m^−1^∙K^−1^, low dielectric constant and low thermal expansion coefficient [11,12]. However, traditional TIMs generally achieve high thermal conductivity due to the addition of a large number of fillers, which not only leads to a higher production cost but also to a decrease in mechanical properties. Therefore, investigating how to carry out the rational design of filler networks to obtain a high thermal conductivity under a relatively low filler fraction has become an important research direction.

In order to achieve this aim, two main strategies have been reported. The first one is to promote the directional arrangement of fillers to build an orderly thermal conductive network using several special methods, such as hot pressing [13,14,15], casting molding [16], ice-template [17,18,19,20], vacuum filtration [21,22,23], electrospinning [24,25,26], etc. Among them, the ice-template method is extremely suitable for the preparation of the TIMs, which benefits from its low processing shrinkage, controllable porosity and superior mechanical strength. For example, Zhang et al. [27] prepared 3D-BNNS/GR/EP by the ice template method, and the composites exhibited superior through-plane thermal conductivities of 2.23 W·m^−1^·K^−1^ at a filler content of 11.2 vol%, demonstrating a 1073% thermal conductivity enhancement at room temperature (RT) compared with pure epoxy resin. Furthermore, adding a small amount of low-dimensional fillers as the second phase is also a common method to achieve high thermal conductivity since nanowires, whiskers and nanosheets with high aspect ratios are highly favorable for constructing fast pathways for phonon transport. To date, BN nanosheets (BNNS) are the most widely used low-dimensional ceramic fillers due to their extremely high aspect ratio and high intrinsic thermal conductivity (~300 W·m^−1^·K^−1^) [28]. Han et al. [29] prepared EP/ANF/BNNS by the unidirectional freezing method, revealing excellent thermal conductivity of 2.41 W·m^−1^·K^−1^ at 14.9 vol% BNNS. However, the strong van der Waals forces between BNNS are not conducive to their uniform dispersion in the organic matrix, which can easily lead to the generation of porosity and an increase in brittleness [30,31]. In addition, these BNNS prepared by physical or chemical stripping methods typically have some drawbacks, such as low yield and small lateral dimensions [32,33,34], requiring a large amount of fillers to achieve high thermal conductivity. However, the inclusion of a large number of fillers will inevitably weaken the mechanical properties of the composite, and the application range is extremely limited. Apart from BNNS, plate-like Al_2_O_3_ [35] and SiC nanowires [36] have also been reported to be added into the dominant fillers as the second phase. Nevertheless, the low intrinsic thermal conductivities of Al_2_O_3_ and SiC (<100 W·m^−1^·K^−1^) still limit their practical application. Therefore, it is necessary to search for novel low-dimensional fillers that can be used for thermal conductive pathway construction.

Si_3_N_4_ nanowires (Si_3_N_4_NWs) have the advantages of high thermal conductivity and a high aspect ratio, which are beneficial for the construction of thermal conductive pathways. For example, Wan et al. [37] prepared the super-flexible PVA/Si_3_N_4_NWs composite films, achieving a high in-plane thermal conductivity of 15.4 W·m^−1^·K^−1^ at a filling fraction of 50 wt%. In addition, the superior insulating and mechanical properties of Si_3_N_4_NWs meet the practical usage requirements of TIMs. From this point of view, Si_3_N_4_NWs are very suitable to be added to composites as a second phase and bridge to enhance the thermal conductivity of the composite. If this filler is used in combination with AlN, which has a significantly higher thermal conductivity, the thermal conductivity of the composite is improved because the degree of interfacial phonon scattering tends to be enhanced during the connection formation process.

Inspired by the considerations mentioned above, in this study, we reported a novel approach for designing a thermal conductive network using AlN particles as the main filler and Si_3_N_4_NWs as the secondary filler. On this basis, we employed the ice template method to rationally create a well-aligned three-dimensional filler skeleton, which was further filled with epoxy resin to obtain polymer-based composites using the vacuum impregnation technique. Due to the enhancement effect of 1D Si_3_N_4_NWs and directionally aligned filler skeletons, the prepared composites exhibited enhanced thermal conductivity. In addition, the introduction of Si_3_N_4_NWs was also favorable in improving the thermal stability and mechanical performance of composites, providing broad application prospects in the field of heat dissipation.

## 2. Materials and Methods

### 2.1. Materials

Silicon powder (Si) and silicon dioxide (SiO_2_) were used as raw materials, and were purchased from Aladdin Reagent Company (Shanghai, China) and Shanghai Sinopharm Holding Company (Shanghai, China), respectively. Carbon paper was used as the growth substrate of Si_3_N_4_NWs and was provided by the Tianjin Carbon Factory. To address gradation considerations, two types of AlN particles with different sizes (H01, ~1 μm, and F80, ~80 μm) were used. H01-AlN was purchased from Tokuyama Co., Japan, and F80-AlN was sourced from Xiamen Ju-Ci Co., Xiamen, China. Tertiary butyl alcohol (TBA) was obtained from Shanghai McLean Biochemical Technology Co. Ltd., Shanghai, China. Lastly, epoxy resin 816 (EP, monomer bisphenol F type) and curing agent 312 (MHHPA) were obtained from Hansen Chemical Co. (Zhuzhou, China) and Guangzhou Hoyun Chemical Co. (Guangzhou, China), respectively.

### 2.2. Process of Preparing Si_3_N_4_NWs

Si_3_N_4_NWs were prepared by a modified carbothermal reduction reaction using a mixture of SiO_2_ and Si powder as the Si source, while carbon paper was implemented as the carbon source and the growth substrate for Si_3_N_4_NWs. Firstly, Si and SiO_2_ were homogeneously ball-milled (200 rpm, 2 h) in a mass ratio of 2:1, and the resulting mixture solution was dried at 80 °C. Carbon paper was folded into a wave shape to serve as a reaction site. The mixed powder was evenly spread on the surface of the carbon paper and then transferred to a tube furnace for a high-temperature reaction. The temperature was increased to 1500 °C at a rate of 5 °C/min and then held for 2 h under flowing N_2_. Then, the furnace was cooled naturally to room temperature. After the reaction, contiguous Si_3_N_4_NWs were grown on the carbon paper substrate and could be easily collected. The obtained Si_3_N_4_NWs were kept in the air at 750 °C for 3 h to remove any residual carbon that might have been present on the surface of Si_3_N_4_NWs.

### 2.3. Preparation of Si_3_N_4_NWs/AlN Directional Skeletons

**Figure 1:**
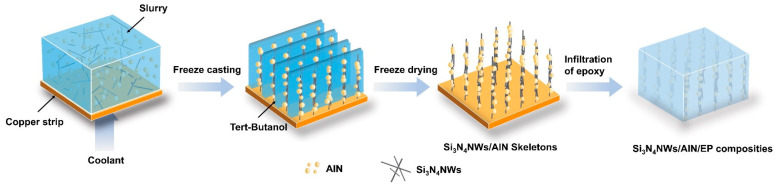
Preparation process of aligned Si_3_N_4_NWs/AlN/EP composites using ice-template method.

The prepared Si_3_N_4_NWs and commercially available AlN were used as thermally conductive fillers, PVB as a binder and EP as the polymer matrix for the thermally conductive composites. The preparation of Si_3_N_4_NWs/AlN/EP directional composites consisted of a four-step process, as illustrated in Figure 1. First, Si_3_N_4_NWs and anhydrous ethanol were configured into a suspension (0.2 g/mL) and then ball-milled at 200 rmp for 1 h. The obtained homogeneous mixed slurry was poured into a Petri dish and then completely dried at 80 °C in an oven. Secondly, Si_3_N_4_NWs and AlN were added proportionally into the TBA solution of PVB, and then the mixing solution was stirred well in a vacuum mixer. During this process, the mass ratio of H01-AlN and F80-AlN was fixed at 1:2, the PVB was added at 0.3 wt% of the total filler amount and the amount of TBA used was related to the filler volume fraction of the EP-base composites in the subsequent steps. Then, the stirred slurry was poured into a polytetrafluoroethylene (PTFE) mold, which was surrounded by PTFE and Cu at the bottom. The molds were placed in a cryogenic reaction tank at −80 °C for 10 min in the direction of the temperature gradient and then freeze-dried at 60 °C for 24 h. After demolding, the AlN/SiN composite skeleton was obtained.

### 2.4. Preparation of the Si_3_N_4_NWs/AlN/EP Composites

Si_3_N_4_NWs/AlN/EP composites were prepared by vacuum-assisted impregnation of epoxy. The epoxy resin and curing agent were homogeneously mixed in a 3:1 mass ratio and vacuum stirred for 30 min. Then, the Si_3_N_4_NWs/AlN skeletons were completely immersed into the mixture under vacuum for 2 h. After that, the samples were poured into silicone molds and cured at 80 °C for 2 h.

In order to investigate the effect of directional networks on the properties of TIMs, we also prepared randomly mixed composites. The epoxy resin and curing agent were mixed homogeneously under vacuum for 30 min. Then, a certain amount of Si_3_N_4_NWs and AlN were added into the polymer and mixed in a vacuum stirrer for 30 min. The mixture was then poured into a silicone mold and cured at 80 °C for 2 h.

Specifically, the Si_3_N_4_NWs/AlN/EP composites prepared by freeze casting and the randomly mixing method were named D-*x*SiN/*y*AlN/EP and R-*x*SiN/*y*AlN/EP, where D and R were abbreviations for directional and random, while *x* and *y* represented the mass fraction of Si_3_N_4_NWs and AlN, respectively.

### 2.5. Characterizations

The phase composition of Si_3_N_4_NWs was performed by X-ray diffraction (XRD, X’Pert PRO MPD, Bruker, Germany) in the range of 10–80° (2θ) using a Cu target. The morphology was characterized by scanning electron microscopy (SEM, Zeiss Gemini 30, Zeiss, Oberkochen, Germany). The thermal conductivity of the composites was determined by a Xi’an Xiaxi electronic hot-wire tester (TC3000E, Xi’an Xiaxi Electronic Technology Co., Ltd., Xi’an, China) with a sample size of 30 mm × 30 mm × 3 mm, and the average value was taken by repeating the test 5 times. Moreover, the universal mechanical testing machine (CMT6103, Sansi Eternal Technology (Zhejiang) Co., Ltd., Taizhou, China) was used to analyze the compressive strength of the composites, and the specific sample dimensions were consistent with that of the thermal conductivity test. Thermal synchronization analysis (TA SDT650) was also used to analyze the thermal stability of composites. Finally, the surface temperature changes were observed using an infrared imager (E6-WIFI, Shenzhen Yujie Hongye Technology Co., Ltd., Shenzhen, China), and the heat dissipation ability of the thermal conductive block in practical use was analyzed.

## 3. Results and Discussion

### 3.1. Characterization of Si_3_N_4_NWs

**Figure 2:**
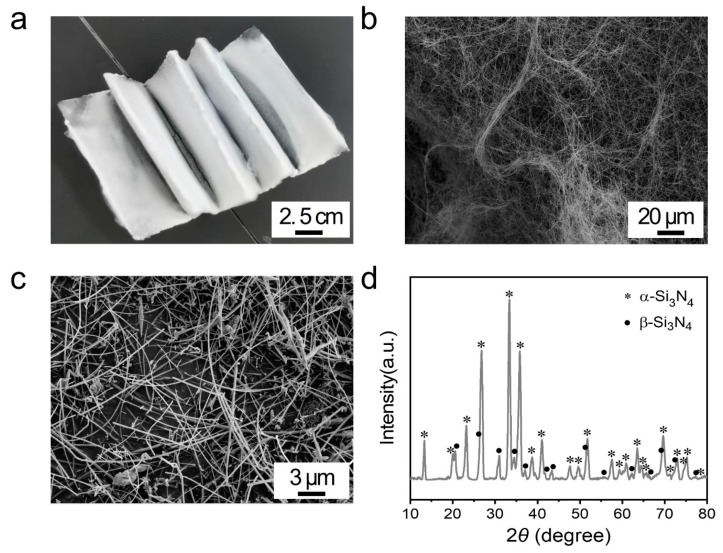
Characterization of the as-synthesized Si_3_N_4_NWs: (**a**) digital picture; (**b**,**c**) SEM images; (**d**) XRD pattern.

Figure 2a shows the macroscopic morphology of Si_3_N_4_NWs generated by the reaction at 1500 °C. It can clearly be seen that the Si_3_N_4_NWs grown on the carbon paper have a cotton-like morphology, which has the advantage of high yield. Figure 2b,c show the SEM images under different magnifications of Si_3_N_4_NWs. It was evident that the Si_3_N_4_NWs prepared by this approach have a high aspect ratio. Moreover, it can be observed from Figure 2c that no small droplet-like particles appeared at the end of the generated nanowires. Considering that no catalyst was added throughout the entire reaction process, it can be inferred that the Si_3_N_4_NWs were formed through the vapor–solid growth mechanism. The XRD pattern of Si_3_N_4_NW is shown in Figure 2d. The nanowires contained two crystalline phases, i.e., the α-phase (PDF NO. 76-1416) and the β-phase (PDF NO. 83-0700), with α-Si_3_N_4_ being the main crystalline phase. No other impurity peaks were detected, suggesting high purity of the synthesized Si_3_N_4_NW. In addition, all the diffraction peaks were sharp, indicating their high crystallinity. Additionally, these high-purity and high-aspect-ratio Si_3_N_4_NWs are very favorable for their subsequent application as thermally conductive fillers.

### 3.2. Characterization of Si_3_N_4_NWs/AlN/EP Thermal Conductivity Skeleton

**Figure 3:**
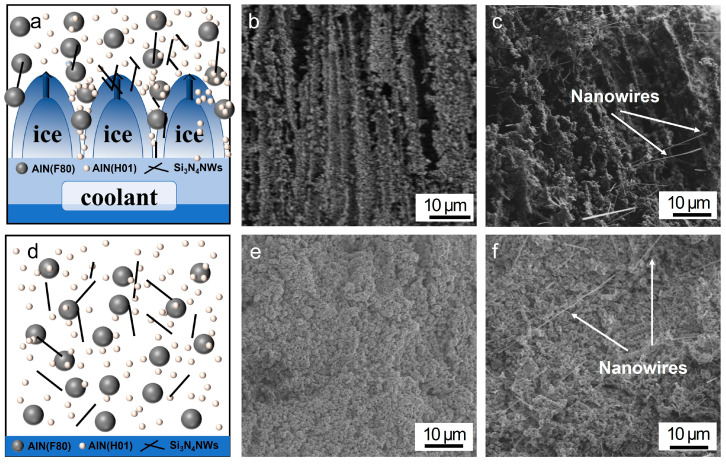
Schematic illustration of the mechanism of the (**a**) ice-template and (**d**) random distribution process. Cross-sectional SEM images of the Si_3_N_4_NWs/AlN skeletons: (**b**) D-55AlN; (**c**) D-1SiN/55AlN; (**e**) R-55AlN; (**f**) R-1SiN/55AlN.

The filler arrangement and the corresponding skeleton morphology in the directionally and randomly distributed composites are represented in Figure 3. As shown in Figure 3a, a unidirectional ice-template technique was used to freeze the suspensions of Si_3_N_4_NWs and AlN. The mold used in the freezing process was composed of PTFE material at the periphery and Cu flakes at the bottom. Due to the large difference in thermal conductivity between Cu and PTFE, there was a significant temperature difference between the samples in the horizontal and vertical directions. As a result, the TBA was preferentially crystallized in the longitudinal direction (i.e., the direction of the temperature gradient), leading to a directional arrangement of Si_3_N_4_NWs and AlN fillers in response to the ice crystal growth forces.

The directional structures of the D-55AlN and D-1SiN/55AlN skeletons are shown in Figure 3b,c. It has been observed that the aluminum nitride particles in both samples were arranged in parallel along the longitudinal direction, indicating that the ice template method was very beneficial for constructing directional thermal conductivity pathways. As for the sample of D-1SiN/55AlN, the Si_3_N_4_NWs were obviously interspersed among AlN fillers, which was beneficial for the rapid directional conduction of phonons and the reduction of thermal resistance. Figure 3e,f show the morphology of the randomly distributed thermally conductive skeletons. As illustrated in Figure 3d, Si_3_N_4_NWs, AlN and EP were prepared by simple blending. Therefore, compared with the directional skeletons, the AlN particles and Si_3_N_4_NWs were randomly aligned, i.e., the thermal conductive pathways were anisotropic. Evidently, the oriented filler skeletons were more favorable for obtaining higher thermal conductivity in the vertical direction [38].

**Figure 4:**
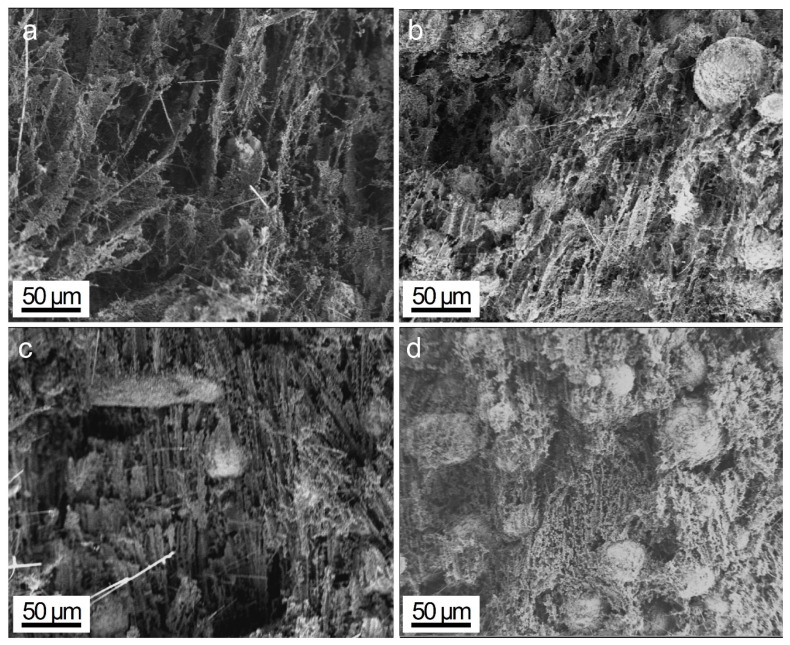
Cross-sectional SEM images of Si_3_N_4_NWs/AlN skeletons containing 1 wt% Si_3_N_4_NWs but with various AlN content: (**a**) D-1SiN-35AlN, (**b**) D-1SiN-45AlN, (**c**) D-1SiN-55AlN, (**d**) D-1SiN-65AlN.

Figure 4 shows the cross-sectional SEM images of the thermally conductive skeleton of 1SiN/*y*AlN, where the loading of AlN filler was progressively increased from 35 wt% to 70 wt% and denoted as D-1SiN/35AlN, D-1SiN/45AlN, D-1SiN/55AlN and D-1SiN/65AlN, respectively. As shown, the morphology of the skeletons mainly depended on the filling fraction of AlN. Overall, at different filling fractions, the AlN particles were directional along the freezing direction, and this special distribution allowed the formation of a directional thermal network between the AlN fillers, which was beneficial in reducing the interfacial thermal resistance. However, as the filling fraction increased, the density of the skeletons gradually increased, the directional effect gradually deteriorated and the horizontal spacing of the vertical thermal pathways gradually decreased. When the filling fraction of AlN was as high as 65 wt%, the directional effect of the thermally conductive skeletons was no longer obvious, and the cross-sectional morphology was similar to an ant’s nest. This phenomenon was mainly due to the decrease of TBA content and the reduction of the driving force of TBA crystallization at high filling fraction, which made it difficult to promote the directional arrangement of a large number of AlN particles at the same time. In addition, it could be noticed that the high-aspect-ratio Si_3_N_4_NWs bridged between AlN particles, which was conducive to shortening the thermal conductivity paths and obtaining higher composite thermal conductivity.

### 3.3. Thermal Conductivity of the Si_3_N_4_NWs/AlN/EP Composites

**Figure 5:**
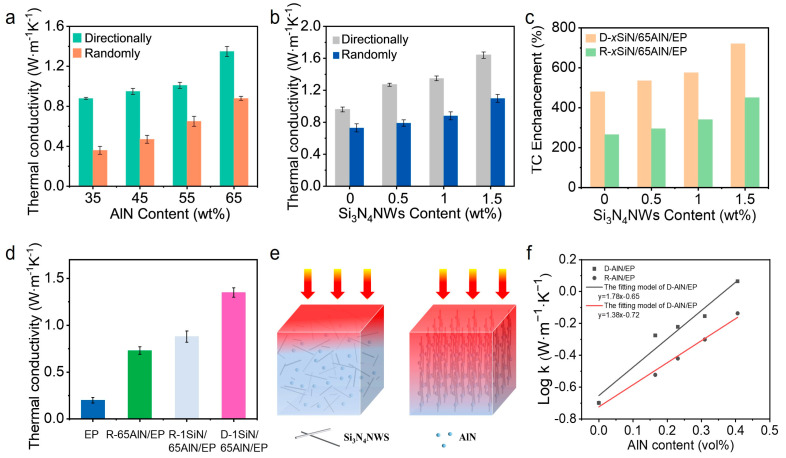
(**a**) Thermal conductivity of 1SiN/*y*AlN/EP composites; (**b**) thermal conductivity and (**c**) *TCE* of *x*SiN/65AlN/EP composites; (**d**) comparison of thermal conductivity between EP and typical *x*SiN/*y*AlN/EP composites; (**e**) illustration of the thermally conductive network formed inside the SiN/AlN/EP composites; (**f**) Agari model simulations.

Figure 5a shows the variation of thermal conductivity with filling fraction for both D-1SiN/*y*AlN/EP and R-1SiN/*y*AlN/EP composites. With the same amount of Si_3_N_4_NWs (1 wt%), the thermal conductivities of the composites showed a continuous increase with the increase of AlN filling fraction, and the R-1SiN/65AlN/EP sample has the highest thermal conductivity of 1.35 W∙m^−1^∙K^−1^. This observation was mainly due to the increase of the internal thermal conduction paths constructed by the AlN particles. In addition to this, the thermal conductivity of the directional composites prepared by the freezing method was also significantly higher than that of the random blends for the same filling fraction. The increase in thermal conductivity was mainly due to the formation of a large number of directional thermal conductive networks and more heat flow channels during the ice templating process, which facilitated phonon transport [39]. In addition, during the TBA freezing crystallization process, a large number of AlN particles accumulated along the longitudinal direction and were in close contact with each other, which further reduced the interfacial thermal resistance between interacting AlN particles.

To further demonstrate the thermal conductivity enhancement of the composites by Si_3_N_4_NWs, the thermal conductivity of the composites with the same percentage of AlN filling (65 wt%) but different Si_3_N_4_NWs addition was analyzed. Figure 5b shows the effect of different preparation processes and Si_3_N_4_NWs additions on the composite thermal conductivity. The oriented samples exhibited higher thermal conductivity than the random sample. In addition, the thermal conductivity was significantly improved with the addition of Si_3_N_4_NWs, and the highest thermal conductivity of 1.64 W∙m^−1^∙K^−1^ was achieved for the sample of D-1.5SiN/65AlN/EP. The experimental results mentioned above indicated that the addition of Si_3_N_4_NWs and the directional pathways provided by the ice templates were beneficial in providing more thermal pathways to enhance the thermal conductivity of the composites [40]. In order to illustrate the enhancement effect of Si_3_N_4_NWs and directional thermal conduction pathways on the thermal conductivity, the thermal conductivity enhancement (*TCE*) of the composites was further calculated with the increase of Si_3_N_4_NWs addition under different preparation processes.

The *TCE* calculation formula is shown in Equation (1):

(1) $$TCE=\frac{k-k_{m}}{k_{m}}$$

where *k* was the thermal conductivity of the composite and *k_m_* was the thermal conductivity of EP. The corresponding calculation results are shown in Figure 5c. The *TCE* increased with the increase of Si_3_N_4_NW content, which indicated that the incorporation of nanowires could significantly improve the thermal conductivity of the composites. The thermal conductivity of D-1.5SiN/65AlN/EP was 1.64 W·m^−1^·K^−1^, which was ~720% of the thermal conductivity of the pure EP (0.2 W·m^−1^·K^−1^). In addition, the thermal conductivity of composite materials prepared by the ice template method was significantly higher than that of randomly mixed samples. For example, the thermal conductivity of D-65AlN/EP was 160% of R-65AlN/EP, which further indicated that the directional thermal conductivity pathway has a significant enhancement effect on the thermal conductivity of composite materials.

Figure 5d provides a further visual comparison of the thermal conductivities of EP, R-65AlN/EP, R-1SiN/65AlN/EP and D-1SiN/65AlN/EP. It was evident that the trend of the thermal conductivity was as follows: EP < R-65AlN/EP < D-65AlN/EP < D-1SiN/65AlN/EP. This trend further suggests that both the construction of directional channels using the ice template method and the addition of Si_3_N_4_NWs could significantly enhance the thermal conductivity of the composites. This enhancement effect is further illustrated in Figure 5e. The directional filler skeletons and the high-aspect-ratio nanowires provided more thermally conductive channels, which was in agreement with previous reports [41,42,43,44].

The Agari model can effectively predict the thermal conductivity of composites at high filling fractions, and it can also be used to determine the construction of internal thermal pathways within the composites. Therefore, the Agari model was utilized to predict the ease of formation of thermally conductive chains within the SiN/AlN/EP composites prepared by different processes.

The Agari model is shown in Equation (2) [45]:

(2) $$\log k=\Phi_{f}\cdot C_{2}\cdot \log k_{f}+(1-\Phi_{f})\cdot \log\left(C_{1}\cdot k_{m}\right)$$

where *k*, *k_f_* and *k_m_* represented the thermal conductivity of the composites, AlN and EP; while the *k_f_* and *k_m_* took the values of 320 W∙m^−1^∙K^−1^ and 0.2 W∙m^−1^∙K^−1^, respectively, in this study. *Φ_f_* was the volume fraction of AlN fillers, *C*_1_ was the parameter of filler influence on the secondary structure of the polymer matrix and *C*_2_ denoted the degree of difficulty in the formation of the thermal network (i.e., the larger the value of *C*_2_, the more complete the thermally conductive network that was formed).

Linear simulation lines were plotted in terms of logarithmic values of filler additions and thermal conductivity of the composites (D-AlN/EP and R-AlN/EP), and the resulting simulation of the Agari model is shown in Figure 5f. Then, the data were substituted into Equation (2), and the values of *C*_1_ and *C*_2_ could be calculated based on the results of the Agari model simulation, where *C*_2_ was calculated as 0.55 for R-AlN/EP and 0.71 for D-AlN/EP. By comparing the *C*_2_ values obtained from the fitting of R-AlN/EP and D-AlN/EP composites, it can be found that the *C*_2_ of D-AlN/EP was larger, indicating that a more complete internal thermal conductivity network was formed [24]. This result further demonstrated that the directional thermally conductive channels constructed by ice templates favored the phonon transfer and further enhanced the thermal conductivity of the composites [46,47].

**Figure 6:**
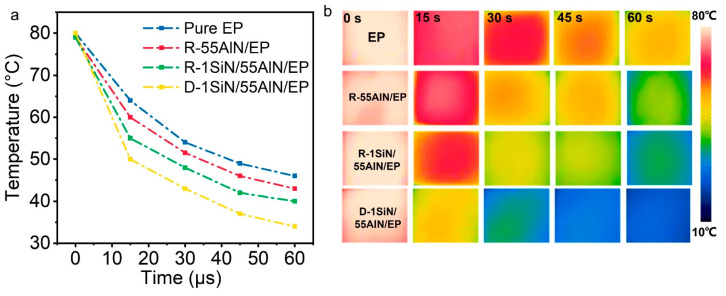
(**a**) Curve of surface temperature change during cooling of heated EP, R-55AlN/EP, R-1SiN/55AlN/EP and D-1SiN/55AlN/EP; (**b**) infrared thermography of EP, R-55AlN/EP, R-1SiN/55AlN/EP and D-1SiN/55AlN/EP.

In order to prove that the SiN/AlN/EP composites can be well applied for thermal management, the actual heat dissipation process of the composites was further explored. Four samples of pure EP, R-55AlN/EP, R-1SiN/55AlN/EP and D-1SiN/55AlN/EP were heated at a constant temperature of 70 °C. When the same temperature was reached, all samples were placed at room temperature at the same time for the natural heat dissipation process. The corresponding temperature changes and the thermal images on the surface of composites were recorded by an infrared camera, as shown in Figure 6a,b, respectively. The cooling rates of different samples were significantly different, and the overall order was as follows: EP < R-55AlN/EP < R-1SiN/55AlN/EP < D-1SiN/55AlN/EP. In addition, the actual trend of temperature changes during the cooling process was consistent with the infrared temperature images. As indicated, the D-1SiN/55AlN/EP composite with both directional thermally conductive skeleton and Si_3_N_4_NWs addition exhibited the best heat dissipation performance, which further illustrated the enhancement of directional thermal conductive pathways and one-dimensional nanowires on the composite thermal conductivity [40].

**Figure 7:**
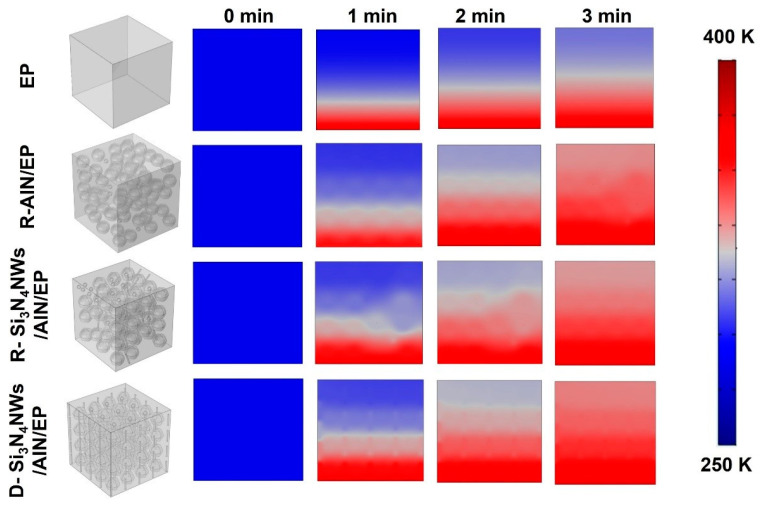
Transient temperature simulation of *x*SiN/*y*AlN/EP composites.

In order to further characterize the contribution of fillers with different morphologies to the thermal conductivity of the composites, we performed transient finite element analysis using the COMSOL Multiphysics v4.4 software to compare and evaluate the heat transfer properties of different composites. As shown in Figure 7, four models were constructed for simulation: pure EP, EP with random distribution of AlN, EP with random distribution of AlN and Si_3_N_4_NWs and EP with oriented distribution of AlN and Si_3_N_4_NWs. Firstly, we created a cube with a radius of 3 cm× 3 cm× 3 cm as the polymer matrix, with an initial temperature set at 293 K. Additionally, the bottom surface of the cube was set as the heat source with a temperature of 343 K. In order to study the heat transfer inside the composites at different time intervals, external natural convection on the solid surface was established for a total duration of 3 min with a time step of 1 min. The simulation results are shown in Figure 7. When no thermally conductive filler was added to the organic matrix, the inherent low thermal conductivity of the matrix led to very slow heat transfer. After adding AlN as a filler into the matrix, the overall thermal conductivity of the composite improved. However, AlN as a filler alone could not effectively form a heat transfer path within the matrix and resulted in significant phonon scattering. When high-aspect ratio Si_3_N_4_NWs were used as fillers and combined with AlN, the thermal conductivity of the composite material increased significantly. On this basis, when the AlN and Si_3_N_4_NWs were further oriented in the vertical direction, the heat transfer rate was further accelerated. This further indicated that the addition of nanowires and the construction of an oriented thermally conductive filler skeleton significantly improved the thermal conductivity, which aligned with the results of thermal conductivity characterization (Figure 5) and thermal infrared imaging (Figure 6).

**Figure 8:**
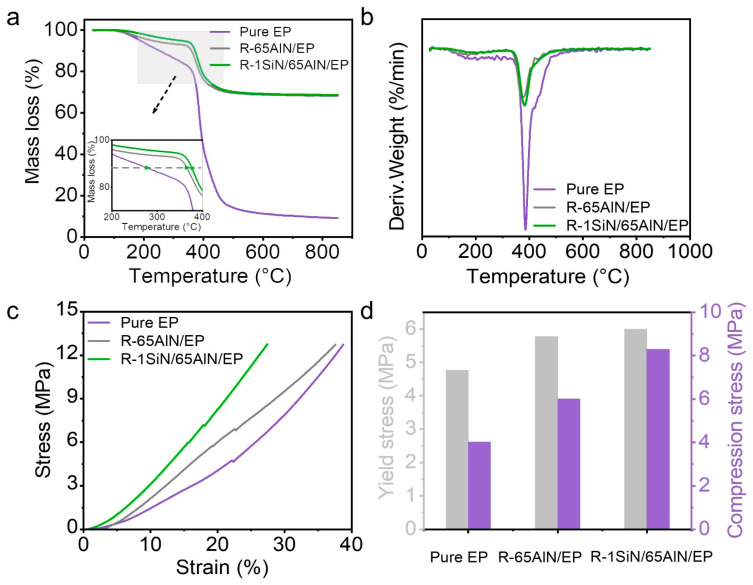
Thermal stability and mechanical performance of EP, R-65AlN/EP and R-1SiN/65AlN/EP: (**a**) TG, (**b**) DTG, (**c**) stress–strain curve and (**d**) yield stress and compressive stress at 20% compressive strain.

Thermal stability and mechanical strength are crucial for thermal management materials. Figure 8a shows the TG curves of the pure EP, R-65AlN/EP and R-1SiN/65AlN/EP, respectively. As observed, both the addition of AlN particles and Si_3_N_4_NWs had an influence on the thermal stability of the composites. For pure EP, the weight loss process mainly occurred in the temperature interval of 200–400 °C, which was due to the decomposition of EP during the heating process. For pure EP, the T10% (the temperature at which 20% of weight was lost) was 360 °C. With the addition of AlN particles and Si_3_N_4_NWs, the T10% increased to 380 °C and 395 °C, respectively. The decomposition temperature of the composites was significantly increased, indicating better thermal stability. This performance was due to the fact that the addition of fillers to the composites affects the crystallinity of the organics, hindering the movement of the organic molecular chains and increasing the thermal decomposition temperature of organics. The DTG curves in Figure 8b indicate that the temperature corresponding to the fastest weight loss of composites increased by the addition of thermally conductive fillers. Particularly, when only 1 wt% of Si_3_N_4_NWs was added, the thermal stability of the composites showed a considerable improvement. The stress–strain curves, which yield stress and compressive strength, of pure EP, R-65AlN/EP and R-1SiN/65AlN/EP are shown in Figure 8c,d. It was indicated that the compressive strength corresponded to the following order: R-1SiN/65AlN/EP > R-65AlN/EP > pure EP. This was mainly due to the high brittleness of the epoxy resin, thus presenting the lowest compressive strength. However, the addition of fillers, especially high-aspect-ratio Si_3_N_4_NWs, could significantly enhance the hardness of the composites, leading to an increase in the compressive strength. The results above strongly indicate that the addition of AlN fillers and Si_3_N_4_NWs could significantly enhance the thermal stability and mechanical strength of the composites, making them better in line the application requirements of the thermal management materials.

## 4. Conclusions

In this study, an oriented AlN/Si_3_N_4_NWs thermal conductive network was successfully constructed by the ice-template method, and the composites were further obtained after vacuum impregnation of epoxy resin. The Si_3_N_4_NWs could effectively bridge between AlN particles, promoting the formation of fast phonon transport channels to improve the thermal conductivity of composites. On this basis, constructing the directional thermal transfer pathways by the ice-template method could increase the composite thermal conductivity from 0.88 W∙m^−1^·K^−1^ for R-1SiN/65AlN/EP to 1.35 W∙m^−1^·K^−1^ for D-1SiN/65AlN/EP. The enhancement effect of Si_3_N_4_NWs and directional thermally conductive pathways on the composite thermal conductivity was also demonstrated by practical heat dissipation applications and finite element simulations. Furthermore, the Si_3_N_4_NWs could not only enhance the heat transfer ability but also improve the thermal stability and mechanical properties of the composites, which further confirmed that Si_3_N_4_NWs had a broad application prospect as fillers for thermal management materials.

## Data Availability

The data presented in this study are available on request from the corresponding author.
